# Predictability of fetal pulmonary artery Doppler on neonatal outcomes in pregnant women with gestational diabetes mellitus

**DOI:** 10.1111/cga.70018

**Published:** 2025-07-10

**Authors:** Huriye Ezveci, Şükran Doğru, Fikriye Karanfil Yaman

**Affiliations:** ^1^ Division of Maternal and Fetal Medicine Necmettin Erbakan University (NEU) Faculty of Medicine Konya Turkey; ^2^ Konya City Hospital Konya Turkey

**Keywords:** acceleration time, ejection time, gestational diabetes mellitus, pregnancy, pulmonary artery Doppler

## Abstract

This study examined the impact of blood glucose‐regulated gestational diabetes (GDM) on fetal pulmonary artery Doppler parameters. This prospective case–control study was performed at a tertiary university hospital. The study cohort comprised GDM patients with controlled blood glucose levels and a healthy control group. Acceleration time/ejection time (At/Et) of the main pulmonary artery, right pulmonary artery, and left pulmonary artery Doppler parameters were assessed and contrasted between the two groups. The study comprised 90 patients, with 30 in the gestational diabetes mellitus group focused on blood sugar regulation and 60 in the healthy control group. No statistically significant difference was observed between the two groups regarding the common main pulmonary artery At/Et (*p* = 0.465), right pulmonary artery At/Et (*p* = 0.237), and left pulmonary artery At/Et (*p* = 0.283). No statistically significant difference was noted between the two groups regarding APGAR scores and blood gas parameters of the newborns (*p* < 0.05). No statistically significant difference was observed in fetal pulmonary artery Doppler parameters (*p* < 0.05) when gestational diabetes mellitus cases were classified based on the utilization of diet and insulin for blood sugar regulation. The pulmonary artery Doppler parameters in fetuses of pregnant women with gestational diabetes mellitus and controlled blood sugar levels are comparable to those of healthy controls. Moreover, dietary habits and insulin administration for glycemic control did not alter pulmonary artery Doppler metrics. The findings suggest that well‐managed gestational diabetes mellitus, irrespective of the treatment modality, do not substantially influence the fetal pulmonary artery dynamics.

## 1 INTRODUCTION

Gestational diabetes mellitus (GDM) is a glucose intolerance condition diagnosed in the second or third trimester in women who are not overtly diabetic before pregnancy and usually resolves after delivery.^1^ Worldwide prevalence varies from 2% to 38% due to differences in population characteristics and choice of screening and diagnostic criteria.^2^ GDM is associated with various adverse outcomes for both mother and fetus, such as macrosomia,^3^ birth trauma,^4^ increased risk of operative and cesarean delivery,^5^ preeclampsia,^6^ metabolic problems in the neonatal period, and neonatal respiratory problems, which are among the serious neonatal complications.^6^, ^7^

Decreased fetal pulmonary artery vascular resistance, tissue impedance, and increased blood flow in the pulmonary vascular bed are important stages in the developmental processes of the fetal pulmonary system.^8^ Any disruption in these developmental stages may adversely affect the maturation of pulmonary vascular structures. Respiratory problems appear to be more common in infants of GDM mothers due to maternal hyperglycemia impairing or delaying surfactant synthesis or due to decreased fluid clearance in the fetal lung.^9^, ^10^

Langer et al. looked at the lecithin/sphingomyelin ratio in amniotic fluid to see how biochemical lung maturation was affecting pregnancies complicated by diabetes mellitus. They found that there was no significant difference in fetal lung profiles between pregnant women with well‐controlled diabetes and women who did not have diabetes.^11^ In another study, De Roche et al. demonstrated that the number of lamellar bodies in the amniocentesis of diabetic pregnant women had high sensitivity and specificity, similar to the lecithin/sphingomyelin ratio, in predicting biochemical fetal lung maturation. They also found that the difference in lung maturation between the well‐controlled diabetic and the control groups was insignificant.^12^

The development of ultrasound technology allows for the indirect evaluation of pulmonary tissues by measuring fetal pulmonary artery Doppler parameters.^13^ In fact, recent studies have found that the amniocentesis method, which is used to invasively assess lung maturity, has a good correlation with the fetal pulmonary artery acceleration time/ejection time (At/Et) measured with a new noninvasive method.^14^ Previous studies have demonstrated that GDM influences fetal vascular resistance, potentially leading to alterations in fetal hemodynamics, particularly affecting the pulmonary circulation.^15^

While the main pulmonary artery (MPA) is commonly used to assess the fetal pulmonary circulation, assessing the right pulmonary artery (RPA) and left pulmonary artery (LPA) separately may reveal segmental variations in vascular resistance that may be masked when assessing only the MPA.^16^ Subtle and regionally variable endothelial changes may also occur in the fetal pulmonary vasculature due to the altered metabolic and inflammatory environments in GDM. Therefore, assessing the MPA, RPA, and LPA separately may allow for a more comprehensive assessment of pulmonary hemodynamics.

This study aims to investigate the effects of GDM on fetal pulmonary artery Doppler parameters with blood glucose regulation. It also aims to investigate the effects of different treatment strategies (diet and insulin therapy) for blood glucose regulation for women with GDM on pulmonary artery Doppler parameters.

## 2 MATERIALS AND METHODS

This prospective study was approved by the Ethics Committee of Necmettin Erbakan University Faculty of Medicine with the decision number 2023/4190 (ID 12949) and was conducted under national legislation, institutional policies, and the Declaration of Helsinki. The study included cases from our tertiary clinic follow‐up between June 1, 2023 and April 1, 2024, that satisfied our study's criteria. After information about the study, informed consent was obtained from all eligible and volunteer participants.

Our inclusion criteria for the study were as follows: pregnant women between the ages of 18–45; singletons and those who gave live birth; gestational diabetes mellitus regulated under diet or insulin treatment; delivery at 37 weeks or more, since the development of the fetal pulmonary vascular system is a dynamic process related to gestational age^17^ and pregnancy follow‐up and delivery should be performed in our clinic. Neonatal respiratory problems are more common in diabetic fetal lungs due to decreased fluid clearance compared with pregnancies without diabetes. Considering that this situation may contribute to the need for more cesarean sections in diabetic pregnancies,^10^, ^18^ cases planned for cesarean section with the indication of a previous cesarean history were included in the case and control groups to ensure homogeneity of the groups.

Exclusion criteria included multiple pregnancies, pregestational diabetes mellitus, unregulated GDM, and preexisting maternal systemic disease. Additionally, fetal growth retardation, known fetal anomaly, PPROM (preterm premature rupture of membranes), premature birth, intrauterine ex fetus, cases with preeclampsia, and patient groups who delivered vaginally were excluded from the study. Cases in which pulmonary artery Doppler measurement could not be performed due to maternal obesity or other reasons were also excluded.

The diagnosis of GDM was made when the threshold values determined in the 75‐g 2‐h Oral Glucose Tolerance Test (OGTT) accepted by the International Association of Diabetes and Pregnancy Study Groups (IADPSG), the American Diabetes Association (ADA),^1^ and the American College of Obstetricians and Gynecologists (ACOG)^19^ were obtained or above these values (the threshold values were as follows: fasting blood glucose 92 mg/dL, 1st hour postprandial value 180 mg/dL, 2nd hour postprandial value 153 mg/dL). This test is performed between 24 and 28 weeks of pregnancy to detect conditions where insulin resistance is significantly increased and the body cannot have the capacity to secrete sufficient insulin to maintain the required glucose levels.

Patients diagnosed with GDM were then followed up by the endocrinology department in terms of blood sugar regulation. Diet was started to provide the glycemic target (fasting blood sugar concentration: <95 mg/dL, one‐hour postprandial blood sugar concentration: <140 mg/dL, two‐hour postprandial glucose concentration: <120 mg/dL),^19^ and insulin treatment was started in cases where diet was not sufficient.

One day before the cesarean section, a single operator (H.E.) conducted this study with a Voluson E8 ultrasound device (the device allows for high‐resolution images with a probe in the frequency range of 2–15 MHz. GE Medical Systems, Tiefenbach, Austria), taking three distinct measurements and calculating their averages. We scanned pregnant women in the supine position, performing imaging only when the fetuses showed no movement or respiration.

**FIGURE 1:**
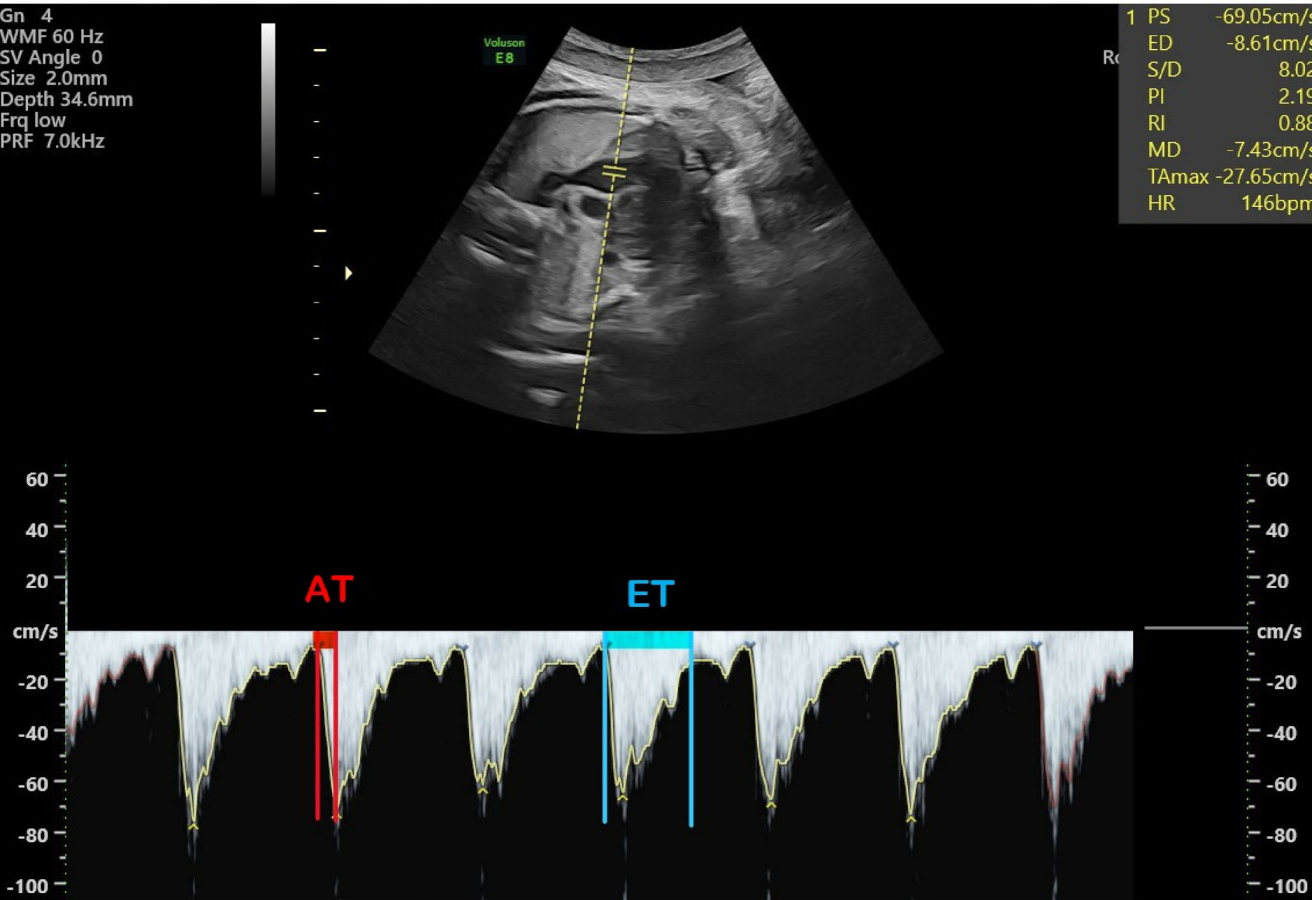
Main pulmonary artery Doppler parameter. AT stands for acceleration time, which is the time between the start of systole and its peak, and ET stands for ejection time, which is the time between the start of systole and its end.

**FIGURE 2:**
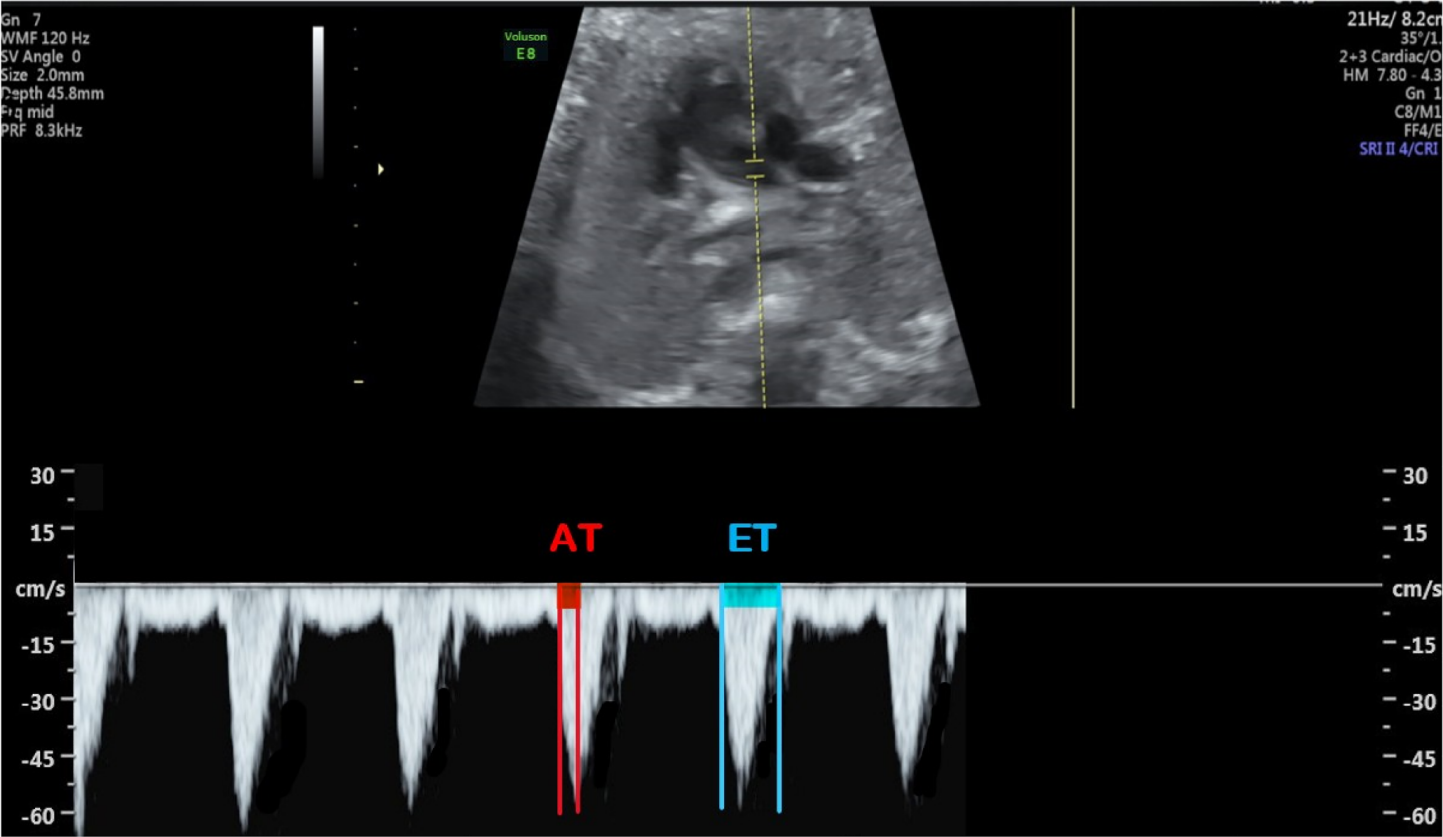
Right pulmonary artery Doppler parameter. AT stands for acceleration time, which is the time between the start of systole and its peak, and ET stands for ejection time, which is the time between the start of systole and its end.

**FIGURE 3:**
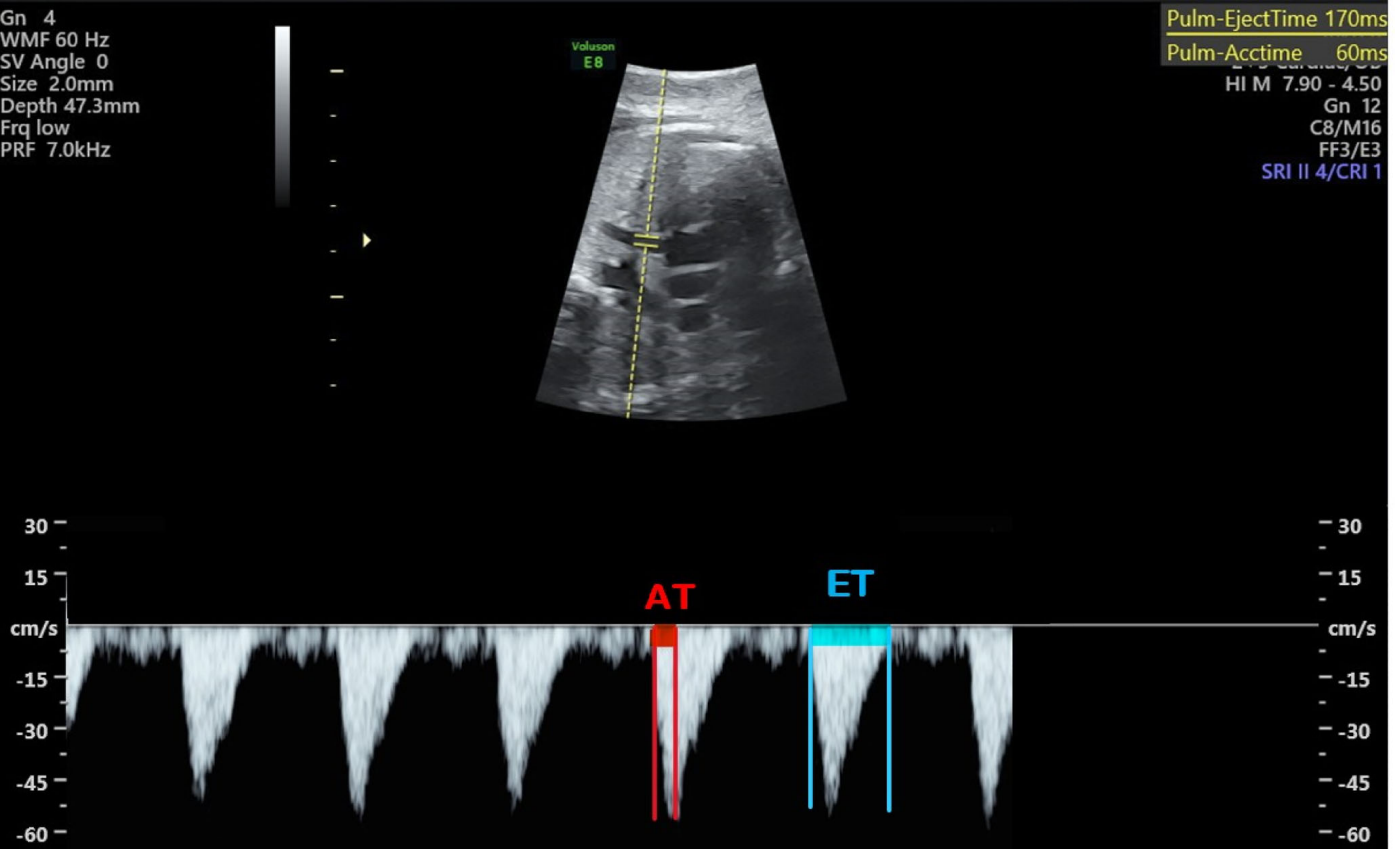
Left pulmonary artery Doppler parameter. AT stands for acceleration time, which is the time between the start of systole and its peak, and ET stands for ejection time, which is the time between the start of systole and its end.

The MPA was followed by the ultrasound probe passing from the four‐chamber view of the heart to the short axis. The MPA valves and the bifurcation of the right and left branches of the pulmonary artery were clearly shown. Doppler sampling was placed in the middle of the fetal MPA, between the pulmonary valve and the artery bifurcation, and was kept away from the arterial walls. We measured the MPA waveform from the main pulmonary artery just distal to the pulmonary valve and obtained the RPA and LPA waveforms from the initial regions of the pulmonary artery bifurcation. After obtaining the best visualization, the sample volume gate was adjusted to 3, and the insonation angle was kept below 20°. The MPA, RPA, and LPA At/Et calculations were used (Figures 1, 2, 3, respectively). AT stands for acceleration time, which is the time between the start of systole and its peak, and ET stands for ejection time, which is the time between the start of systole and its end.

Following the cesarean section, we clamped the umbilical cord and collected umbilical venous blood samples from 15 cm away from the placenta into a heparinized syringe. We recorded the pH, partial oxygen (pO_2_), carbon dioxide (pCO_2_), bicarbonate, lactate, and base minus of the umbilical venous blood. We delivered the newborn baby to the neonatal team.

## 3 STATISTICAL ANALYSES

The normality of the data distribution was assessed using the Kolmogorov–Smirnov test, Shapiro–Wilk test, and histograms. For continuous variables that followed a normal distribution, an independent *t*‐test was performed, and the results were presented as mean ± standard deviation or median (minimum–maximum). For continuous variables that did not follow a normal distribution, the Mann–Whitney *U* test was used, and the results were presented as mean ± standard deviation. All statistical tests were two‐tailed, and a significance level of *p* < 0.05 was considered statistically significant. Data were analyzed using Statistical Package for Social Sciences (SPSS) software, version 20.

## 4 RESULTS

This prospective study consisted of 90 cases, 30 well‐controlled GDMs, and 60 controls. The mean age of the GDM group (33) was slightly higher than the control group (29). However, this difference was not statistically significant (*p* = 0.059). The mean birth weight of the GDM group was 3352 ± 459 g, while it was 3165 ± 360 grams in the control group. The birth weight of the GDM group was significantly higher than the control group (*p* = 0.038). There was no significant difference between the groups in the APGAR scores at 1 and 5 min (*p* = 0.822 and *p* = 0.269). There were no significant differences between the GDM and control groups in terms of umbilical venous blood gas parameters (*p* > 0.05). Neither group experienced NICU admission or neonatal respiratory problems (Table 1).

**TABLE 1:** Demographic and clinical characteristics of blood sugar regulated gestational diabetes mellitus (GDM) patients and control group.

Parameters	GDM (*n* = 30)	Control (*n* = 60)	*p*‐value
Age	33 (23–45)	29 (19–43)	0.059^a^
Gestational week	38 (37–39)	38 (37–40)	0.252^a^
Birth weight	3352 ± 459	3165 ± 360	0.038^a^
1 min APGAR	6 (5–8)	6 (4–8)	0.822^a^
5 min APGAR	7 (5–9)	7 (6–9)	0.269^a^
Umbilical venous blood			
pH	7 ± 0	7.04 ± 0.12	0.242^a^
pO_2_ (mmHg)	29.8 ± 10.5	28.3 ± 7.9	0.610^a^
pCO_2_ (mmHg)	37.9 ± 9.3	39.8 ± 6.5	0.464^a^
HCO_3_ (mEq/L)	20.5 ± 3.7	22.2 ± 2.2	0.065^a^
Lactate (mmol/L)	2.2 ± 1.1	1.7 ± 0.7	0.097^a^
Base excess (mmol/L)	−3.8 ± 3.2	−2.3 ± 1.8	0.109^a^
NICU admission	0	0	

Abbreviations: APGAR, Activity and muscle tone, Pulse (heart rate) Grimace response (medically known as “reflex irritability”) Appearance (skin coloration) Respiration; HCO_3_, bicarbonate; NICU, neonatal intensive care unit; pH, potential of hydrogen; pCO_2_, partial carbon dioxide pressure; pO_2_, partial oxygen pressure.

^a^ Independent *t*‐test.

MPA At/Et, RPA At/Et, and LPA At/Et values were measured as 0.31 ± 0.14 and 0.33 ± 0.13; 0.31 ± 0.08 and 0.34 ± 0.12; 0.39 ± 0.15 and 0.36 ± 0.11 in the well‐controlled GDM and control groups, respectively, and these differences were not taken into account (*p* = 0.465, *p* = 0.237, and *p* = 0.283, respectively) (Table 2).

**TABLE 2:** Fetal pulmonary artery Doppler in blood sugar regulated gestational diabetes mellitus (GDM) and control group.

Parameters	GDM (*n* = 30)	Control (*n* = 60)	*p*‐value
MPA AT/ET	0.31 ± 0.14	0.33 ± 0.13	0.465^a^
RPA AT/ET	0.31 ± 0.08	0.34 ± 0.12	0.237^a^
LPA AT/ET	0.39 ± 0.15	0.36 ± 0.11	0.283^a^

Abbreviations: AT, acceleration time, which is the time between the start of systole and its peak; ET, ejection time, which is the time between the start of systole and its end; LPA, left pulmonary artery; MPA, main pulmonary artery; RPA, right pulmonary artery.

^a^ Independent *t*‐test.

Fetal pulmonary artery parameters of GDM cases were compared between diet and insulin‐regulated groups in Table 3. When MPA At/Et, RPA At/Et, and LPA At/Et values were compared between diet‐regulated and insulin‐regulated groups, they were measured as 0.29 ± 0.1 and 0.33 ± 0.18; 0.31 ± 0.08 and 0.32 ± 0.09; 0.40 ± 0.16 and 0.37 ± 0.13, respectively, and these differences were not found to be statistically significant (*p* = 0.632, *p* = 1, *p* = 0.983).

**TABLE 3:** Comparison of fetal pulmonary artery parameters between diet‐regulated and insulin‐regulated gestational diabetes mellitus cases.

Parameters	Diet (*n* = 18)	İnsulin (*n* = 12)	*p*‐value
MPA AT/ET	0.29 ± 0.1	0.33 ± 0.18	0.632^a^
RPA AT/ET	0.31 ± 0.08	0.32 ± 0.09	1^a^
LPA AT/ET	0.40 ± 0.16	0.37 ± 0.13	0.983^a^

Abbreviations: AT, acceleration time, which is the time between the start of systole and its peak; ET, ejection time, which is the time between the start of systole and its end; LPA, left pulmonary artery; MPA, main pulmonary artery; RPA, right pulmonary artery.

^a^ Mann–Whitney *U* test.

## 5 DISCUSSION

In our study, we observed no difference in pulmonary artery Doppler parameters (MPA At/Et, RPA At/Et, LPA At/Et) between the fetuses of pregnant women with GDM who had blood sugar regulation and delivered after the 37th week and the fetuses in the healthy control group who delivered at the same gestational week. Furthermore, the provision of blood sugar regulation through diet or insulin did not significantly alter pulmonary artery parameters.

In 2017, Büke et al. showed that MPA At/Et values were negatively associated with respiratory distress syndrome (RDS) in fetuses of pregnant women who gave birth before 37 weeks.^20^ In 2018, the same researchers measured the MPA At/Et values of 87 pregnant women who did not have congenital fetal abnormalities, chronic maternal diseases, and pregnancy complications (gestational diabetes, preeclampsia, etc.) and gave birth after 34 weeks. In the study, cases diagnosed with transient tachypnea (TTN) in newborns and those not diagnosed were divided into two groups, and the MPA At/Et values between the two groups were compared. As a result of the examination, despite the adjustments made according to gestational age and newborn weight, they found a negative correlation between MPA At/Et values and TTN.^21^ However, the non‐TTN group used 18.6% of steroids, while the TTN‐diagnosed group did not use any steroid treatment. Steroid treatment may accelerate lung maturation, which could potentially influence the results. The study also did not take into account the type of delivery or the reason for delivery, so it is possible that these factors, which may have an impact on the incidence of TTN, were not taken into account. This could link MPA At/Et and TTN less clearly. In 2020, Duncan et al. examined MPA At/Et values in cases between 23 and 36 weeks of gestation complicated by preterm prelabor rupture of membranes (PPROM) and stated that PPROM did not increase the prognostic accuracy, but MPA At/Et was considered a strong predictor.^22^ In their prospective study of pregnant women with confirmed SARS‐CoV‐2 infection at 32–39 weeks of gestation, Göncü et al. observed NICU admission only in the SARS‐CoV‐2‐positive group and stated that MPA At/Et values were lower in this group, but this finding may be due to differences in gestational weeks.^23^

Another study from 2022 found that fetuses in pregnancies complicated by intrahepatic cholestasis (IHCP) had MPA At/Et values that were significantly higher than in healthy pregnancies, but there was no correlation between NICU admission, respiratory distress, and MPA At/Et.^24^

In a previous study, the waveforms of the pulmonary arteries in the fetuses of pregnant women diagnosed with GDM were examined, and the relationship between these parameters and respiratory disorders (NRD) in newborns was investigated. In these studies, it was found that the decreases in At/Et in the left and right peripheral pulmonary arteries were significantly associated with NRD rates and could predict NRD. However, this relationship was not observed with the MPA, RPA, and LPA At/Et results.^25^ We can conclude from these findings that GDM induces microvascular effects on the fetal peripheral pulmonary vessels, leading to changes in their resistance levels. However, the study did not provide details on how blood sugar regulation in GDM may affect these results.

Han et al. divided the GDM cases and healthy controls with controlled blood sugar into 8 time periods ranging from 31 weeks to 38 + 6 weeks according to gestational age. They evaluated each group, 15 well‐controlled GDM and 25 controls, according to the fetal lung development by week, MPA At/Et, and lung development diameters. The research found a link between MPA At/Et and gestational age, but there was no significant difference in pulmonary artery Doppler parameters between the well‐controlled GDM and control groups over the 8 time periods.^15^ This study did not examine the right or left pulmonary artery Doppler parameters or control pregnant women with GDM using diet or insulin.

We found no significant difference in pulmonary artery Doppler parameters between the GDM and healthy control groups in our study. Furthermore, neither diet nor insulin regulation of blood sugar affected these parameters. This finding suggests that GDM treatment does not have a significant effect on fetal pulmonary vascular development, at least in parameters that can be monitored with Doppler ultrasound. No NICU admissions or neonatal respiratory problems were observed in either the study group or the control group. This result is supported by the secondary finding of the recent Antenatal Late Preterm Steroids (ALPS) study, which indicated that 306 (10.8%) of 2831 women had gestational diabetes mellitus (GDM), with no clinically significant difference in neonatal respiratory disorders.^26^ It is also good evidence and a result of the cooperation of perinatology, endocrinology, and neonatology.

This study has several limitations. The sample size was relatively small, potentially limiting the ability to detect subtle differences in pulmonary artery Doppler parameters. The study also lacked long‐term follow‐up to evaluate the effects of GDM and its management on respiratory health. The study exclusively examined Doppler ultrasound measurements and did not evaluate alternative imaging techniques or biomarkers related to fetal lung development, which could offer additional insights into the impact of gestational diabetes mellitus on fetal respiratory function.

## 6 CONCLUSION

This study found no significant differences in pulmonary artery Doppler parameters between fetuses of well‐controlled GDM pregnancies and healthy controls, suggesting that well‐controlled GDM may not significantly affect fetal pulmonary vascular development.

## CONFLICT OF INTEREST STATEMENT

The authors have no conflicts of interest.

## ETHICAL STATEMENTS

This prospective study was approved by the Ethics Committee of Necmettin Erbakan University Faculty of Medicine with decision number 2023/4190 (ID 12949) and was conducted under national legislation, institutional policies, and the Declaration of Helsinki.
